# Prevention of mother-to-child HIV-1 transmission in Burkina Faso: evaluation of vertical transmission by PCR, molecular characterization of subtypes and determination of antiretroviral drugs resistance

**DOI:** 10.3402/gha.v8.26065

**Published:** 2015-01-27

**Authors:** Tani Sagna, Cyrille Bisseye, Tegewende R. Compaore, Therese S. Kagone, Florencia W. Djigma, Djeneba Ouermi, Catherine M. Pirkle, Moctar T. A. Zeba, Valerie J. T. Bazie, Zoenabo Douamba, Remy Moret, Virginio Pietra, Adjirita Koama, Charlemagne Gnoula, Joseph D. Sia, Jean-Baptiste Nikiema, Jacques Simpore

**Affiliations:** 1 Biomolecular Research Centre Pietro Annigoni (CERBA/LABIOGENE), University of Ouagadougou, Burkina Faso, West Africa; 2 Institute of Research in Health Sciences, IRSS, Bobo-Dioulasso, Burkina Faso; 3 Laboratory of Molecular and Cellular Biology, University of Sciences and Techniques of Masuku (USTM), Franceville, Gabon; 4 Saint Camille Medical Centre, Ouagadougou, Burkina Faso; 5 Population Health and Optimal Health Practices Research Unit, CHU de Québec Research Centre, Quebec, Canada

**Keywords:** pregnant women, HAART, sequencing, genotypes, mutations

## Abstract

**Background:**

Vertical human immunodeficiency virus (HIV) transmission is a public health problem in Burkina Faso. The main objective of this study on the prevention of mother-to-child HIV-1 transmission was to determine the residual risk of HIV transmission in infants born to mothers receiving highly active antiretroviral therapy (HAART). Moreover, we detect HIV antiretroviral (ARV) drug resistance among mother–infant pairs and identify subtypes and circulating recombinant forms (CRF) in Burkina Faso.

**Design:**

In this study, 3,215 samples of pregnant women were analyzed for HIV using rapid tests. Vertical transmission was estimated by polymerase chain reaction in 6-month-old infants born to women who tested HIV positive. HIV-1 resistance to ARV, subtypes, and CRFs was determined through ViroSeq kit using the ABI PRISM 3,130 sequencer.

**Results:**

In this study, 12.26% (394/3,215) of the pregnant women were diagnosed HIV positive. There was 0.52% (2/388) overall vertical transmission of HIV, with rates of 1.75% (2/114) among mothers under prophylaxis and 0.00% (0/274) for those under HAART. Genetic mutations were also isolated that induce resistance to ARV such as M184V, Y115F, K103N, Y181C, V179E, and G190A. There were subtypes and CRF of HIV-1 present, the most common being: CRF06_CPX (58.8%), CRF02_AG (35.3%), and subtype G (5.9%).

**Conclusions:**

ARV drugs reduce the residual rate of HIV vertical transmission. However, the virus has developed resistance to ARV, which could limit future therapeutic options when treatment is needed. Resistance to ARV therefore requires a permanent interaction between researchers, physicians, and pharmacists, to strengthen the network of monitoring and surveillance of drug resistance in Burkina Faso.

Globally, about 35.3 million people were living with HIV in 2012. Sub-Saharan Africa is the region most affected by the HIV epidemic with approximately 25 million people living with the virus (1). However, since 2001, the annual number of new HIV infections among adults has declined by 34%. In Burkina Faso, the number of people living with HIV decreased from 2001 to 2012 (180,000 vs. 110,000). In this country the number of pregnant women living with HIV is less than 2% (2) and the antiretroviral (ARV) prevention services for pregnant women living with HIV had a coverage of about 50–79% in 2012 (1). Highly active antiretroviral therapy (HAART) has reduced significantly the mortality rate in patients infected with HIV (3). However, it increased the diversification of ARV-resistant strains of HIV and their distribution worldwide.

In the early 2000s, like other countries in sub-Saharan Africa, Burkina Faso launched a national program for the prevention of mother-to-child transmission of HIV and Saint Camille Medical Centre in Ouagadougou was the first operational pilot site (4, 5). This program offered screening and HIV treatment to pregnant women and their household (spouse, children, co-wives) through the prevention of mother-to-child HIV transmission (PMTCT) approach recommended by WHO at the time (6). In 2013, the WHO issued new consolidated guidelines (B+ option) for use of ARV drugs and prevention, recommending lifelong initiation of antiretroviral therapy (ART) for all pregnant and breastfeeding women living with HIV, regardless of their T CD4 cell counts (1). However, Burkina Faso is still in the early stages of implementing the new WHO guidelines. The 2010 version of the WHO guidelines, on the use of ARV for PMTCT, were based on the need to distinguish between offering HAART treatment for the mother's health versus prophylaxis (7).

Despite its effectiveness, HIV vertical transmission prevention and ARV prophylaxis could contribute to the selection of resistant viral strains (8, 9). Indeed, in Burkina Faso, Nadembega et al. (10) and Simpore et al. (5, 11) have already emphasized the context by which PMTCT led to resistance mutations induced by nevirapine (NVP; V8IV, K103N, V179E, and Y181C). In 2012, WHO estimated that the prevalence of ARV resistance among pregnant women in Burkina Faso was between 5 and 15% and indicated that these frequencies were the highest among those observed in Africa (12).

In this study, we first evaluate the results of the new mother-to-child HIV transmission prevention protocol by estimating the residual rate of vertical transmission of HIV. Second, we identify resistant strains to ARV drugs and determine the subtypes and circulating recombinant forms (CRF) of HIV-1 in Burkina Faso.

## Methods

### Study subjects

From October 2009 to June 2013, 3,215 pregnant women with less than 32 weeks of amenorrhea attended a prenatal consultation at Saint Camille Medical Centre (CMSC, Ouagadougou, Burkina Faso) and agreed to answer a questionnaire and receive counseling for voluntary HIV testing. This medical center has high attendance, mostly from women with low incomes/assets and/or who already know their HIV serology, because the center delivers low cost medical care and laboratory analyses to HIV-positive pregnant women and their families. Pregnant women attended CMSC until delivery and those newborns positive for HIV-1 despite having followed the PMTCT protocol were included in this study. Pregnant women who did not consent to participate were not included in this study.

### Prophylaxis and HAART

Following WHO recommendations (13), pregnant women with a CD4 count less than 350 cells/µL, regardless of their clinical stage, underwent HAART – triple therapy combining two nucleoside reverse transcriptase inhibitors (NRTI) (AZT/3TC or D4T/3TC) and one NNRTI (NVP) or one protease inhibitor (PI) (LPV_rtv or IDV_rtv) – whereas those with a CD4 count above 350 cells/µL underwent WHO prophylactic protocol (AZT from the 28th week of pregnancy, AZT+3TC+NVP during labor and AZT/3TC during the first week postpartum). Efavirenz (EFV) being an embryotoxic molecule was not used in pregnant women.

### Sampling and sample treatment

Venous blood (6 mL) was collected at the laboratory of Saint Camille Medical Centre on two EDTA-treated tubes and plasma was collected after centrifugation at 4,000 g for 10 min; 1.5 mL aliquots were stored at −80°C. Molecular tests were conducted at the Biomolecular Research Center Pietro Annigoni (CERBA) in Ouagadougou.

### HIV serology

Serology of HIV testing among women was determined using two rapid tests: Determine^®^ (Abbott Laboratories, Tokyo, Japan) and SD Bioline (Standard Diagnostics, Inc., Korea). When the two tests had discordant results in an individual, a third confirmatory test was recommended immediately, according to the national algorithm, with another kit such as Immunocombs (ImmunoComb^®^II HIV-1&2 Bispot, Orgenics, Yavne, Israel).

Infants born to mothers infected with HIV were tested 6 months after birth through a PCR (polymerase chain reaction) of HIV diagnosis test on dried blood spot (DBS). Newborn samples detected as positive were immediately sent to another laboratory for confirmation. Meanwhile, children detected as negative for HIV were followed until the age of 18 months.

For children diagnosed HIV-1 positive, sequencing was carried out from a venous blood sample after serological confirmation test at 18 months.

### CD4 and viral load testing

CD4 cells were counted for mothers using FACSCount techniques and reagents (Becton Dickinson, San Jose, CA). Patient whole blood was homogenized, 50 µL was collected and placed in each tube of the pair of reactive CD4/CD8. Once all the samples were distributed in pairs of reagents corresponding to each patient, they were incubated in the dark for 1 h. After incubation, 50 µL of a fixative solution was added to each tube and then passed through the FACScount machine.

Women HIV Viral loads were determined using the ABBOTT kit and a Real Time PCR M2000 instrument (ABBOTT).

### DNA extraction and detection of HIV-1 proviral DNA

DNA was extracted from DBS using the QIAamp DNA Blood Mini Kit (QIAGEN, Hilden, Germany) following the manufacturer's instructions.

Detection of HIV-1 proviral DNA was carried out in the Applied Biosystems Gene Amp^®^ PCR System 9700 using the kit ‘Generic HIV DNA Cell’ (Biocentric, Bandol, France) following the manufacturer's protocol.

We used 20 µL of extracted DNA and 50 µL of the Master Mix containing PCR water (2 µL), primers and probe (1 µL), 2×mix (25 µL). The amplification was carried out following these steps: Initial activation for 2 min at 50°C, 10 min at 95°C and a denaturation for 15 sec at 95°C, annealing for 1 min at 60°C during 50 cycles. Afterwards, 10 µL of the amplified DNA mixed with 2 µL of running buffer was migrated on electrophoresis (2% agarose gel, 1X TBE and 5 µL of ethidium bromide). The UV revelation was done using the Gene Flash Sygene Bio Imaging device.

### RNA extraction, sequencing test and determination of ARV drugs resistance

Patients selected for the determination of possible resistance to ARV drugs were the women who, despite ARV treatment, had a CD4 count of <350 cells/µL, a high viral load, or who had transmitted HIV-1 to their children or the children tested positive for HIV-1.

Ribonucleic acid (RNA) was extracted from plasma using the QIAamp Viral RNA Mini Kit (QIAGEN Hilden, Germany) according to the manufacturer's instructions. Reverse transcription of RNA into cDNA, and DNA amplification and sequencing were performed using the complete ViroSeqTM Pack (HIV-1 Genotyping System v2.0, Abbott, Santa Clara, USA) according to the manufacturer's instructions. The reverse transcriptase (RT)/PCR product was quantified by electrophoresis on an agarose gel at 1% and purified with ExoSAP-IT (USB Affymetrix^®^ Products, Inc., Cleveland Ohio, USA). After purification of sequence reactions with isopropanol, the sequencing process was started using the ABI PRISM 3,130 Genetic Analyzer (Applied Biosystems, Foster City, CA, USA).

### Statistical analyses

The demographic and clinical data were entered and analyzed using SPSS version 12 for Windows and the EpiInfo Version 6.04.d. The basis of statistical significance was set at *p*<0.05 throughout Student's *t*-test and Fisher Exact tests.

### Ethical aspects

The internal ethics committee of the Saint Camille Medical Centre and the CERBA approved this study and each mother gave her written consent for the collection of blood. A PMTCT code was assigned to each woman adhering to counseling. Blood sampling from a child was done with the consent of one parent. The reference number of ethical clearance certificate was n°2009CR/IV-15/136, ‘Comité d’Éthique Institutionnel du 15/04/2009’.

## Results

### Baseline characteristics of pregnant women

The pregnant women in this study were aged between 18 and 46, with an average age of 26.7±5.6. Among them, there were housewives (54.5%), women working in the informal sector such as trading activities (26.7%), employees (7.6%), high school students (6.8%), and university students (4.5%). Most of the seropositive pregnant women were in their fourth pregnancy or more (33.50% ≥4 pregnancies vs. 12.69%<4 pregnancies: *p*<0.0001). However, among HIV-negative pregnant women, many were in their first pregnancy (38.36% vs. 21.91%: *p*<0.0001).

Pregnant women who had a history of abortions/miscarriages and child deaths were mostly in the group of housewives and the informal sector. These survey results are shown in Table 1.

**Table 1:** Baseline characteristics of pregnant women according to occupational status

Characteristics (medium values)	Employed (1)	University student (2)	High school student (3)	Informal sector (4)	Housewives (5)	Total
	244 (7.6%)	144 (4.5%)	218 (6.8%)	858 (26.7%)	1,751 (54.5%)	3,215
Age	29.67±5.06	25.68±2.54	22.31±3.28	26.72±5.36	26.83±5.86	26.7±5.6
Pregnancies	2.22±1.196	1.38±0.877	1.29±0.641	2.48±1.553	2.82±1.767	2.51±1.652
Abortions/Miscarriages	0.21±0.522	0.21±0.756	0.05±0.268	0.19±0.550	0.19±0.572	0.19±0.558
Deceased children	0.12±0.341	0.02±0.143	0.06±0.256	0.24±0.580	0.35±0.808	0.27±0.686
HIV negative	218/244 (89.34%)	140/144 (97.22%)	213/218 (97.71%)	773/858 (90.09%)	1,477/1,751 (84.35%)	2,821/3,215 (87.74%)
HIV positive	26/244 (10.66%)	4/144 (02.78%)	05/218 (2.29%)	85/858 (9.91%)	274/1,751 (15.65%)	394/3,215 (12.26%)

Pregnancies: *p*<0.0001; abortions/miscarriages: *p*<0.01; deceased children: *p*<0.01.

### HIV prevalence among pregnant women

Among the 3,215 pregnant women who were tested for HIV, 394 (12.26%) were infected and among them 97.97% women had HIV-1, 1.78% had HIV-2, and 0.25% had coinfection of HIV-1/2. But from 2009 to 2013, HIV prevalence decreased (21.43% vs. 10.46%). Table 2 indicates that HIV prevalence increased with age: It varied from 2.81% (age group below 19) to 22.51% for women older than 35 years (*p*<0.001).

**Table 2:** HIV status of pregnant women according to age group

Age groups (years)	HIV serology			HIV type		

	Number	HIV−	HIV+	HIV-1	HIV-2	HIV-1/2
1 (≤19)	285/3,215 (8.90%)	277/285 (97.19%)	08/285 (2.81%)	08/285 (2.81%)	0/7 (0.00%)	0/1 (0.00%)
2 (20–24)	967/3,215 (30.10%)	914/967 (94.52%)	53/967 (5.48%)	53/967 (5.48%)	0/7 (0.00%)	0/1 (0.00%)
3 (25–29)	1,015/3,215 (31.60%)	894/1,015 (88.08%)	121/1,015 (11.92%)	120/1,015 (11.82%)	1/1,015 (0.10%)	0/1 (0.00%)
4 (30–34)	606/3,215 (18.80%)	471/606 (77.72%)	135/606 (22.28%)	134/606 (22.11%)	1/606 (0.17%)	0/1 (0.00%)
5 (≥35)	342/3,215 (10.60%)	265/342 (77.49%)	77/342 (22.51%)	71/342 (20.76%)	5/342 (1.46%)	1/342 (0.29%)
Total	3,215/3,215 (100%)	2,821/3,215 (87.74%)	394/3,215 (12.26%)	386/3,215 (12.01%)	7/3,215 (0.22%)	1/3,215 (0.03%)

*P* (1), (2), (3), (4)<0.001; *P*(4)→(5)=0.93.

### Mother-to-child transmission of HIV

Among the 394 pregnant women who tested HIV positive, six of them were lost to follow up and 388 babies were born. A total of 29.38% children were born to mothers under the WHO 2006 prophylactic protocol (AZT from the 28th week of pregnancy, AZT+3 TC+NVP during labor, AZT/3TC during the first week of postpartum) and 70.62% were born to mothers on HAART (triple therapy combining AZT/3TC+NVP or D4T/3TC+NVP or 2NRTI+1NNRTI or 2NRTI+ 1PI). The median viral load of mothers whose children tested positive for HIV-1 was 161,231 copies per mL, with mean T CD4 cell counts of 185 cells per µL. The tests for the mothers in our study revealed that transmission was higher among women with low rate of CD4 count and high viral load (Table 3). With regard to breastfeeding, 19.3% of women with triprophylaxis breastfed their children for the first 4 months before starting artificial feeding (Table 4). Those with HAART bottle-fed their children exclusively.

**Table 3:** Average CD4 count and median viral load of mothers compared with their children RT**/**PCR of HIV results

RT/PCR results (number)	CD4/µL	Viral load/mL
Positive (2)	185±101	161,231.00
Negative (386)	440±209	37.71
Total (388)	426±212	73.00

CD4 or viral load: RT/PCR positive→negative: *p*<0.05.

**Table 4:** Distribution of women according to ARV, feeding option, and children PCR results

Mothers			Children	

Treatments	Feeding option	Number	PCR negative 386/388 (99.48%)	PCR positive 2/388 (0.52%)
PP^1^ 114/388 (29.38%)	Bottle feeding	92/114 (80.70%)	91/92 (98.91%)	01/92 (1.09%)
	Breastfeeding	22/114 (19.30%)	21/22 (95.45%)	01/22 (4.55%)
HAART^2^ 274/388 (70.62%)	Bottle feeding	274/274 (100%)	274/274 (100%)	0/274 (0.0%)

*P*(1→2)=0.086. PP: prophylactic protocol; HAART: tritherapy associating AZT/3TC+NVP or D4T/3TC+NVP or 2NRTI+1NNRTI or 2NRTI+1PI. From WHO Ref. [13].

Vertical transmission was very low in our study (0.52%). Among mothers under HAART, there were no cases of HIV transmission, but at the level of triprophylaxis, 1.75% cases of transmission have been reported (Table 4).

### Subtypes of HIV-1 infection in mothers and their children

Sequencing was performed for 17 patients, including 11 mothers who had followed the PMTCT protocol and six of their children all infected with HIV-1: Four HIV-positive children were taken from the sample of a previous study, two were from mothers who transmitted HIV to their children in this study. These children's mothers were selected in addition to five other mothers with therapeutic failure.

In this study, 17 samples were sequenced. Recombinant forms between subtypes A and G of HIV-1 were present in 94.1% cases with: 58.8% of CRF06_CPX and 35.3% CRF02_AG. Subtype G was also isolated in 5.9%. The detected subtypes were the same within each mother–child pair (M44/E44: CRF06_CPX; M102/E102: CRF02_AG). The various subtypes obtained from the present study in comparison with those of later studies are described in Table 5.

**Table 5:** Major HIV-1 subtypes and CRFs detected among pregnant women and children in Burkina Faso

	Present study; *n*=17 (Ouagadougou) (%)	Kagoné et al. (28); *n*=46 (Bobo-Dioulasso) (%)	Nadembega et al. (10); *n*=29 (Ouagadougou) (%)	Ouédraogo et al. (27); *n*=70 (Ouagadougou) (%)
CRF06_CPX	58.8	54.5	55.2	50.0
CRF02_AG	35.3	38.6	31.0	30.0
G	5.9		3.5	7.1
A		2.3	6.9	10.0
CRF09_CPX			3.4	
CRF01_AE		4.6		
Others				2.9
Total	100	100	100	100

### HIV-1 mutations causing resistance to ARVs in pregnant women and their children

The ratio of resistance to ARV drugs generated from the ViroSeq software (Abbott, USA) showed the presence of numerous mutations that can cause HIV resistances to ARV drugs (Table 6). Important changes were also found in the RT, which, according to the software used, could lead to HIV resistances.

**Table 6:** Frequency of identified mutations inducing resistance for ARVs used in the prevention of mother-to-child transmission protocol at Saint Camille Medical Centre in Ouagadougou

Major mutations	Numbers	Frequency	ARVs used in PMTCT affected by resistance
M184V	5	18.53	3TC, FTC
K103N	4	14.83	NVP, DLV, EFV
A98G	2	7.41	NVP, EFV
T69S	2	7.41	
E138A	2	7.41	ETR
V179E	2	7.41	NVP, EFV, ETR
Y181C	1	3.70	NVP, EFV, ETR
G190A	1	3.70	NNRTI
Y115F	1	3.70	ABC, TDF
D67R	1	3.70	
D67E	1	3.70	
L74V	1	3.70	DDI, ABC
M184I	1	3.70	3TC, FTC
V90I	1	3.70	ETR
K103E	1	3.70	
G190R	1	3.70	
Total	27	100%	

ARV: antiretroviral; PMTCT: prevention of mother-to-child transmission; 3TC: lamivudine; FTC: emtricitabine; NVP: nevirapine; DLV: delavirdine; EFV: efavirenz; ETR: etravirine; ABC: abacavir; TDF: tenofovir; DDI: didanosine.

## Discussion

The present research work was conducted at Saint Camille Medical Centre in Ouagadougou and is similar to those conducted by Simpore et al. (5, 14). The originality of this work compared to previous studies is highlighted by: 1) The diagnostic tool. Previous studies have used RT-PCR for the detection of viral RNA while this one tested for the presence of proviral DNA by PCR; 2) Protocol. A new protocol was introduced for the PMTCT in conformity with the WHO 2006 instructions; 3) ARV. In this study, new molecules of ARV, less toxic than D4T, were introduced; 4) CD4 counts. The threshold for CD4 count was increased from 200 to 350 cells/µL for the patients to receive treatment. The differences between our study and previous ones are described below.

Regarding the screening of HIV in this study, 12.26% of pregnant women (394/3,215) were diagnosed HIV positive. This rate, although high, is lower than 17.7% identified at the same site by Pignatelli et al. (15) but higher than the prevalence of 11.2 and 7.3% obtained by Simpore et al. (5, 14). An even lower prevalence of 1.9 and 2.62% were found in Democratic Republic of Congo and Cameroon, respectively (16, 17). However, HIV prevalence decreased from 2009 to 2013 (21.43% vs. 10.46%). The national HIV prevalence is less than 2% (2). These variations may be due to the high attendance at the Saint Camille Medical Centre of patients who often already know their HIV status, but come because this center delivers affordable care and laboratory analysis to HIV-positive pregnant women and their families.

In this study, despite the monitoring and treatment of these women during pregnancy, residual risk of transmission to infants born to mothers receiving PMTCT was very low, estimated at 0.52% in total, or 1.72% with prophylaxis and 0.00% with HAART. The global transmission rate obtained in this study is relatively low compared to rate of 4.6% obtained in Cameroon (17). In Burkina Faso, a high level of vertical HIV transmission rate (10.4%) has previously been reported (14). The residual rate of vertical transmission of 0.52% showed that the new prophylactic protocol and the less restrictive criteria for the use of HAART for pregnant women contributed in decreasing the risks of mother-to-child transmission of HIV. Also, Carmone et al. found that infants born to women with more time on ART were less exposed (18). The feeding option could also be a risk factor in our study group; especially because of various cultural or social reasons, some women theoretically use artificial feeding, but also give breast milk to their children (19). It is estimated that half of all new episodes of HIV transmission to children occur during the breastfeeding period when the majority of lactating women are not receiving the prophylaxis necessary to prevent HIV transmission (1).

Late treatment is also a factor which increases the risk of transmission (20). There is still the need to better understand if these women were really receiving their first HIV-positive test, as we discussed above, in relation to the high attendance in the center of this study. Nevertheless, it is likely that some women are infected with a resistant virus and that it is not due to previous exposure to ARV.

In addition to coinfections (21, 22) and late treatment (20), the emergence of resistant HIV strains to ARV drugs resulting in high viral load is also an important factor in HIV vertical transmission. Furthermore, although the sample size is limited, in our study we found that the transmission rate is higher among women with a low rate of CD4 and high viral load.

Many mutations can cause HIV-1 resistance to some ARV drugs, for example, the RT inhibitors used in the context of PMTCT in Burkina Faso. In fact, we have found the mutations to be M184V, K103N, and Y181C. Simpore et al. (5) showed that, in addition to the mutations in the RT (Y18CY), other major protease mutations such as V8IV induced a selection of resistant viral strains. Moreover, an identical mutation (Y181C) found by Nadembega et al. (10) has been isolated in the present study. Several other studies reported that K103N was highly prevalent among NNRTI-associated resistance mutations (23–25) . K103N mutation causes high resistance to NVP and EFV (26), as found in our study, although we have not been able to sequence all our samples. These drugs are mostly used in first line treatment through combination drugs.

Beyond the toxicity and mutations induced by the combined use of ARVs, HAART has reduced not only the number of mutations and resistance but also the residual risk of HIV transmission to infants born to mothers receiving PMTCT.

Moreover, the presence of 5.9% of HIV-1 subtype G was noted in this study, with a total lack of subtypes A or B. However, previous studies in Burkina Faso found subtype A at varying rates: 10.0% (27), 6.9% (10), and 2.3% (28) (Table 5). Recombinant forms between subtypes A and G were identified with proportions that are almost similar to the other studies conducted previously in Burkina Faso.

Thus, CRF06_CPX and CRF02_AG are responsible for a great number of infections in West Africa: In Mali (29): CRF02_AG, 69.6% and CRF06_CPX, 8.7%; in Togo (30): CRF02_AG, 51.2% and CRF06_CPX, 12.5%; in Senegal (31): CRF02_AG, 55.0%, and CRF06_CPX, 7.0%. In Burkina Faso, the predominant form is the CRF06_CPX followed by CRF02_AG (5, 10, 28). Although we were not able to sequence all samples, these analyses of CRF allow us to suggest that CRF06_CPX are part of a chain of transmission in Burkina Faso because of being the predominant subtypes.

## Conclusions

In this study, it appears that pregnant women under HAART have no risk of vertical transmission of HIV-1. This provides evidence of the effectiveness of the new PMTCT program in Burkina Faso.

The CRF CRF06_ CPX and CRF02_AG of HIV-1 are the main strains of infection in our study group. Certainly, ARV treatment significantly reduces vertical transmission of HIV-1. However, it causes mutations that induce resistance of HIV to ART and thus limits the available choice of best drug combination. The real fight against resistance to ARV therefore demands a permanent interaction between researchers, physicians, and pharmacists. Finally, the strengthening of a network of monitoring and surveillance of drug resistance in Burkina Faso is needed.
